# Modification of Frictional Properties of Hydrogel Surface via Laser Ablated Topographical Micro-Textures

**DOI:** 10.3390/nano12224103

**Published:** 2022-11-21

**Authors:** Zhuangzhuang Zhou, Yihang Chu, Zhishan Hou, Xiaopeng Zhou, Yu Cao

**Affiliations:** 1 International Science and Technology Cooperation Base for Laser Processing Robot, Zhejiang Provincial Key Laboratory of Laser Processing Robot, Wenzhou University, Wenzhou 325035, China; 2 School of Optical and Electronic Information, Huazhong University of Science and Technology, Wuhan 430074, China; 3 Oujiang Laboratory (Zhejiang Lab for Regenerative Medicine, Vision and Brain Health), Wenzhou University, Wenzhou 325000, China

**Keywords:** polyvinyl alcohol hydrogel, micro-pit, functional design, surface friction property

## Abstract

Hydrogels and biological cartilage tissues are highly similar in structure and composition due to their unique characteristics such as high-water content and low friction coefficients. The introduction of hydrogel cartilage can effectively reduce the friction coefficient and wear coefficient of the original bone joint and the implanted metal bone joint (generally titanium alloy or stainless steel), which is considered as a perfect replacement material for artificial articular cartilage. How to accurately regulate the local tribological characteristics of hydrogel artificial cartilage according to patient weight and bone shape is one of the important challenges in the current clinical application field of medical hydrogels. In this study, the mechanism by which micro-pits improve the surface friction properties was studied. Ultraviolet lasers were used to efficiently construct micro-pits with different shapes on a polyvinyl alcohol hydrogel in one step. It was shown that by using such a maskless laser processing, the performance of each part of the artificial cartilage can be customized flexibly and effectively. We envision that the approach demonstrated in this article will provide an important idea for the development of a high-performance, continuous and accurate method for controlling surface friction properties of artificial cartilage.

## 1. Introduction

Friction is a kind of universal phenomenon, which produces a force that inhibits the tendency of motion. Modifications of frictional properties via the topographically surface texturing have been studied for years. The most general textures are of two types: micro-dimples (i.e., square, triangular, circular, ellipse, chevron [1]) and micro-grooves (i.e., parallel grooves, hexagonal groovescross-hatched patterns [2]). Vladescu et al. concluded that the effectiveness of grooves perpendicular to the direction of sliding is the best and the chevron pattern is the next followed by the grooves parallel to the sliding direction whose effectiveness is the lowest [3]. Sperka et al. noticed that the increase in the angle of the contact groove to the rolling speed direction has a negative effect on film thickness via experiments [4]. Yu et al. have proven that aspect ratio (AR) has the greatest influence on friction control of groove surface texture [5]. Common surface texture technologies include electric discharge machining [6], laser texturing [5,7], chemical or electrochemical etching [8], micro-electrochemical [9] and mechanical machining methods [10]. In these methods, laser surface texturing has great development prospects due to its unique characteristics of a non-contact process, the lack of a mechanical cutting force, no tool wear [11], CAM automation [12], and accurate control of geometric shape and depth [13].

Hydrogel is a kind of hydrophilic gel which consists of plenty of water and cross-linking networks. Since hydrogels have similar tribological properties to those of natural cartilage, they have become ideal substitutes for articular cartilage, and their bioengineering applications have been widely investigated. With the rapid development of intelligent machines, some mechanical structures in soft robotics and smart actuators require the frictional contact of hydrogels [14] to implement specific functions, such as hydrogel fingers for grasping small balls [15], a soft walking robot [16], and contact within hydrogel bionic arm joints [17]. Therefore, understanding the contact tribological response of hydrogels is crucial for operational accuracy of smart mechanical structures, the underlying mechanism of which is directly related to the failure of such structures and even the success of their tasks [18]. Friction between hydrogels and solid surfaces is complex and does not obey the Amonton’s Law [19]. The friction of hydrogels depends primarily on their chemical structure relative to the surface properties of substrates and the influence of the test environment. According to the classic repulsion–adsorption model proposed by Gong et al. [20,21], when the relative sliding velocity of the hydrogel-solid interface is much slower than characteristic velocity, friction originates from elastic deformation of the hydrogel adsorbed on the substrate and from lubrication of the hydration layer; when the sliding velocity of the interface is much faster than characteristic velocity, a polymer will not have sufficient time to form an adsorption point, and therefore friction will be determined by the hydration layer between the interfaces. Urueña et al. have measured the friction of polyacrylamide hydrogels using a Gemini contact [22]. At low velocities, the law of friction change with velocity contradicts the classic Stokes curve and increasing the cross-linked-mesh size or reducing the polymer concentration can reduce the friction coefficient.

Whether with regard to hard or soft materials, the surface topography plays an important role in the regulation of friction. The regulation of friction for hydrogels by means of random roughness or regular micro-protrusion surfaces has been investigated comprehensively. Both approaches produce interface micro-channels similar to drainage channels; therefore, the fluid between interfaces cannot bear pressure effectively [23]. Nonetheless, tribological properties of hydrogels, particularly those with surface micro-structures, require further research [3], since the micro-structure of the hydrogel surface is often formed simultaneously during the hydrogel forming stage. Friction cannot be precisely regulated in the same or different planes of one piece of hydrogel.

In this study, polyvinyl alcohol (PVA) hydrogels containing surface micro-pit structures of different shapes was prepared in a single step using an ultraviolet (UV) laser. Unlike mold forming, noncontact direct laser processing of the hydrogel surface enabled coplanar profiled preparation. We then explored the mechanisms of surface friction between the hydrogels carrying micro-pits of different shapes and a titanium alloy as well as friction between hydrogels. We established a model for the regulation of tribological properties of a hydrogel surface containing micro-pits of different shapes within stable external-friction pairs. An efficient, continuous, and accurate method for controlling the frictional characteristics of hydrogels was developed, which is also expected to lay the foundation for the design of high-performance and high-compatibility artificial joints.

## 2. Materials and Methods

### 2.1. Frictional Pair Materials

The toxicity and cleaning procedures of PVA to human cells, which are the prerequisite for all medical implants, have been proven by many researchers. For example, Lei Liu and Menghe Zhu, et al. [24] were the first to propose the concept of a dynamic hydrogen-bond nanoconfinement, and the design of highly stretchable and supratough biocompatible PVA with well-dispersed dynamic nanoconfinement phases induced by hydrogen-bond (H-bond) crosslinking. At the end of their paper, the biocopatibility of as-developed PVA/HCPE nanocomposites is assessed in light of their promising applications of as artificial ligaments, the results showing that neither PVA nor PVA/HCPE is highly toxic to humans. 

PVA hydrogels are mainly prepared by physical cross-linking and chemical cross-linking methods, among which the most common physical cross-linking method is the repeated freeze-thawing method. Its main principle is that after repeated freezing and thawing several times, some of PVA physical and mechanical properties can be greatly improved. Freezing makes the molecular chains of PVA in aqueous solution "freeze" in the motion state at a certain time, and the molecular chains in contact can interact with each other and become entangled. Through physical effects such as van der Waals force and hydrogen bonding, the molecular chains are closely combined and cannot be separated in a certain micro-zone, becoming "entangled nodes". During refreezing, new ordered micro-regions are formed, which are called "physical cross-linking points". The freezing-thawing method can promote the movement of molecules, rearrange, and obtain semi-crystalline or crystalline hydrogels through the folding of molecular chains.

PVA (degree of polymerization: 2600; saponification rate: 99%) was purchased from Shandong Yousuo Chemical Technology Co., Ltd. (Qingdao, China). The PVA hydrogel was formed by freeze–thawing a 12% (*w*/*w*) aqueous PVA solution. The aqueous polymer solution was prepared by continuous stirring at 90 °C for 1 h and then poured into customized cylindrical acrylic molds to form smooth hydrogel disks (diameters: 25 and 45 mm; thickness: 3 and 1 mm). After the PVA solution in the molds was subjected to three freeze–thaw cycles via freezing at −40 °C for 16 h and thawing at 21 °C for 8 h, the hydrogels were kept in distilled water for at least 1 week to reach swelling equilibrium.

The compression modulus was assessed by dynamic mechanical analysis (Q800, TA Instruments, New Castle, DE, USA). Samples of 20 mm in diameter and 3 mm in thickness were evaluated at a compression rate of 10% thickness/min. The stress–strain curves showed nonlinear variation primarily due to slight deformations of the hydrogel and variation in the height of natural samples [25]. The relationship between stress and ~9% strain was linear, and the elastic modulus of the hydrogels (638 kPa) was calculated from the slope under these conditions.

Titanium alloy of Chinese brand TC4 (Ti-6Al-4V/Grade 5 in US brand) was used in this work. TC4 is a kind of α-β two-phase titanium alloy which contains 6%wt. aluminium as the α-phase stable element and 4%wt. vanadium as the β-phase stable element. Due to its excellent mechanical properties and good bio-compatibility, TC4 alloy material has been successfully applied in aerospace, medical, chemical and other fields. Titanium alloy disks with a diameter of 45 mm and a thickness of 1 mm (Baoji Bo Yu Xin Industry and Trade Co., Ltd., Baoji, China) were polished using a polishing agent with a particle size of 1 µm and were employed in a friction pair with hydrogels. 

Surface morphology of the friction pair consisting of the hydrogel and titanium alloy was examined under an OSL4100 confocal laser scanning microscope (Olympus Optical Co., Ltd., Tokyo, Japan). The depth values at all levels of textured hydrogels were the average of micro-pits measurements of four morphologies. A specific method for confocal laser scanning microscope to calculate micro-pit depth are provided in Materials and Method section the Supplementary Materials.

Static contact angles (CAs) of samples in 2 µL of distilled water were measured and photographed using an OCA15EC contact angle meter (DataPhysics Instruments GmbH, Filderstadt, Germany). The contact angles of PVA hydrogel and titanium alloy are 9.8° and 96.7°, respectively.

**Figure 1:**
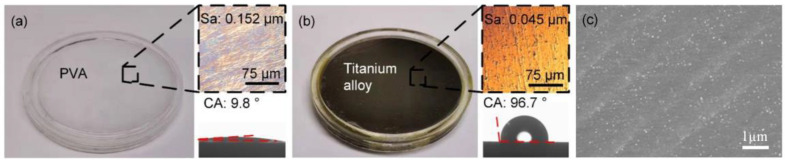
Confocal maps of the smooth titanium alloy and of the hydrogel as well as smooth surface morphology. Surface morphology of (**a**) the hydrogel and (**b**) the titanium alloy. (**c**) the SEM of polished titanium alloy.

Figure 1 indicates that the surfaces of the hydrogel (Sa: 0.152 µm) and the titanium alloy (Sa: 0.045 µm) were randomly rough. Interestingly, the surface of the former was superhydrophilic, whereas that of the latter was hydrophobic. Smooth hydrogel surfaces were used not only as friction substrates but also to create micro-pits on these surfaces.

### 2.2. Laser Surface Texturing 

**Figure 2:**
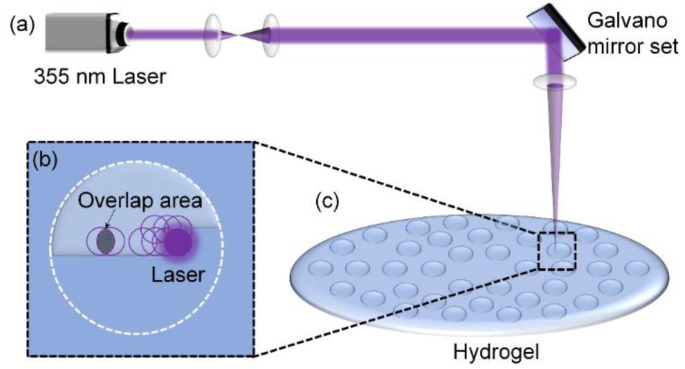
Schematics of the UV laser and of micro-pit array processing. (**a**) UV laser processing equipment, (**b**) Circular micro-pit processing, (**c**) Circular pit distribution.

Hydrogel samples were etched in ambient air (Figure 2a) using a Super Pulse 355-12 ultraviolet laser (Suzhou Yinggu Laser Co., Ltd., Suzhou, China) with the parameters listed in Table 1. To produce regular high-precision micro-pit planar patterns, laser scanning was executed line by line using an x–y Galvano scanner (Figure 2b). Laser power density was 10.2 J/cm^2^, the spot area overlap rate was 84.1%, and scan spacing (d: 10 μm) was smaller than the spot diameter (Φ: 20 μm). The high spot overlap rate and small scan spacing ensured a clearer pattern outline. The micro-pits along the radial direction were spaced at intervals of 400 μm. As the radius increased, the number of micro-pits gradually increased along the circumferential direction (Figure 2c).

### 2.3. Characterization of Surface Tribological Properties

**Figure 3:**
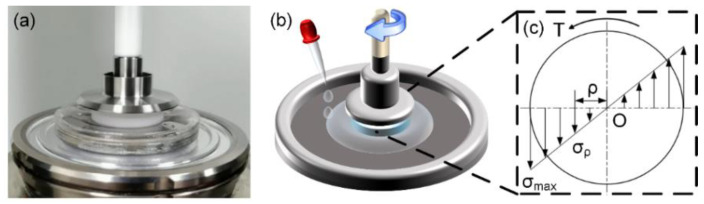
Schematics of the friction test and shear stress distribution. (**a**) The friction test device. (**b**) The schematic of the friction test device. (**c**) Frictional stress distribution. ρ, radial distance; σ_ρ_, frictional stress at a radial distance of ρ; σ_max_, maximum frictional stress; T, friction torque.

Figure 3 presents a hydrogel disk (diameter, 25 mm; thickness, 3 mm) attached to an acrylic disk of the same size with a cyanoacrylate instant adhesive (Xi’an Jianghang Rubber Co., Ltd., Xi’an, China). The detailed process of friction test is provided in Materials and Method section the Supplementary Materials.

Figure 3c depicts the shear stress distribution. Although the velocity between the friction interfaces varied along the radial direction, we assumed that frictional stress (σ) was maximal when relative sliding velocity of the friction interfaces was maximal (*v* = *ωR*). Maximal frictional stress was calculated [26] as:

(1) $$\sigma (\omega R)=2T(\omega )/(\pi R^{3})$$

where the friction torque $T\left(\omega \right)$ measured rheometrically was generated by friction, ω represents angular velocity of the hydrogel surface, and R is the radius of the hydrogel disk.

## 3. Results and Discussion

### 3.1. Topology of the Laser-Textured Hydrogel 

**Figure 4:**
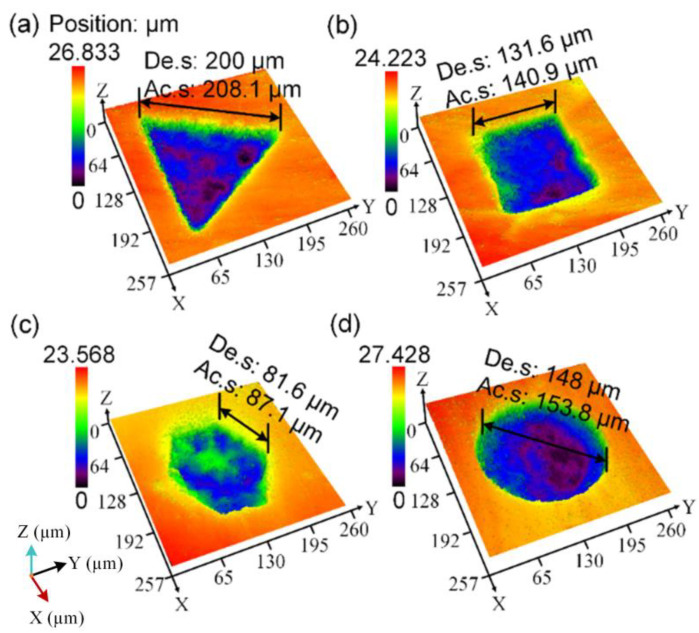
Confocal analysis of the morphology of micro-pits with different shapes. (**a**) Triangular. (**b**) Quadrilateral. (**c**) Hexagonal. (**d**) Circular. Abbreviations: De. s, design size; Ac. s, actual sample size.

Figure 4 shows the micro-pits of various shapes prepared on the hydrogels. Bound water in the PVA hydrogel was converted to free water in pits located in the hydrogel groove after laser etching owing to the destruction of the reticular structure of the hydrogel by the laser. Thus, laser energy absorption causes thermal evaporation of the water at the bottom of the laser ablation micro-pit, and this further leads to a decrease of the surface tension in water and the ionization induced water evaporation [27]. This process was conducive to the removal of hydrogel and formed irregular pits approximately 2 μm deep at the bottom of micro-pits. For the micro-pits of various shapes, the average depth are 24.91 µm (Triangle), 24.65 µm (Square), 24.76 µm (Hexagon) and 25.76 µm (Circle), respectively.

### 3.2. Micro-Pit Wear and a Model of the Wear Mechanism

**Figure 5:**
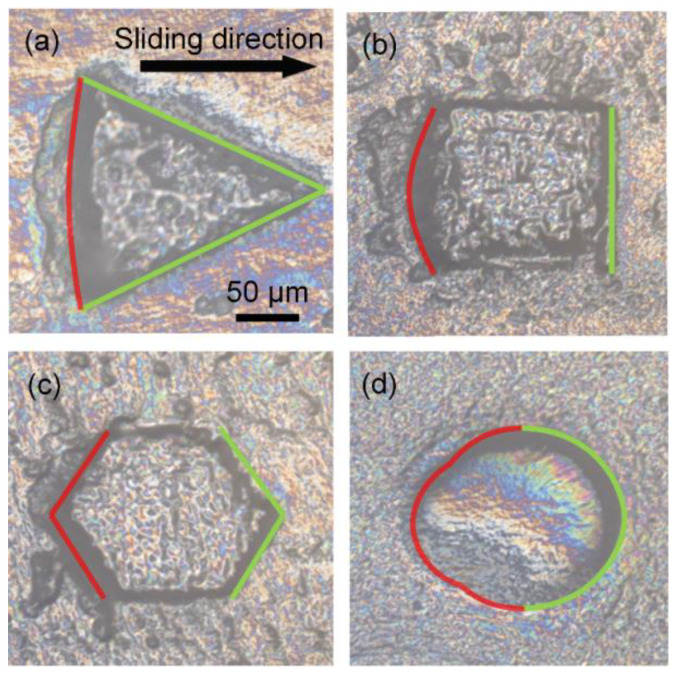
Confocal images of wear. (**a**) Triangle. (**b**) Square. (**c**) Hexagon. (**d**) Circle. The green curve is the leading edge. The red curve is the trailing edge.

In order to better understand the friction mechanism of the hydrogels, we investigated the mechanism underlying wear of the micro-structures by measuring wear on the micro-pits of four shapes under stress (P = 2 kPa) at a velocity of 77.4 mm/s for 2.5 h, with the titanium alloy surface as the substrate (Figure 5). Wear was evident along the trailing edges of all micro-pits but not at the top. The triangular and quadrilateral trailing edges were perpendicular to the sliding direction, and wear was significant in all trailing edges. The hexagonal and circular trailing edges had a specific angle in accordance with the sliding direction, and wear was significant only near the symmetry axis of the trailing edge. In addition, in contrast to general solid wear, all the micro-pits regardless of shape stretched to different degrees along the sliding direction and contracted perpendicular to the sliding direction after the friction test.

**Figure 6:**
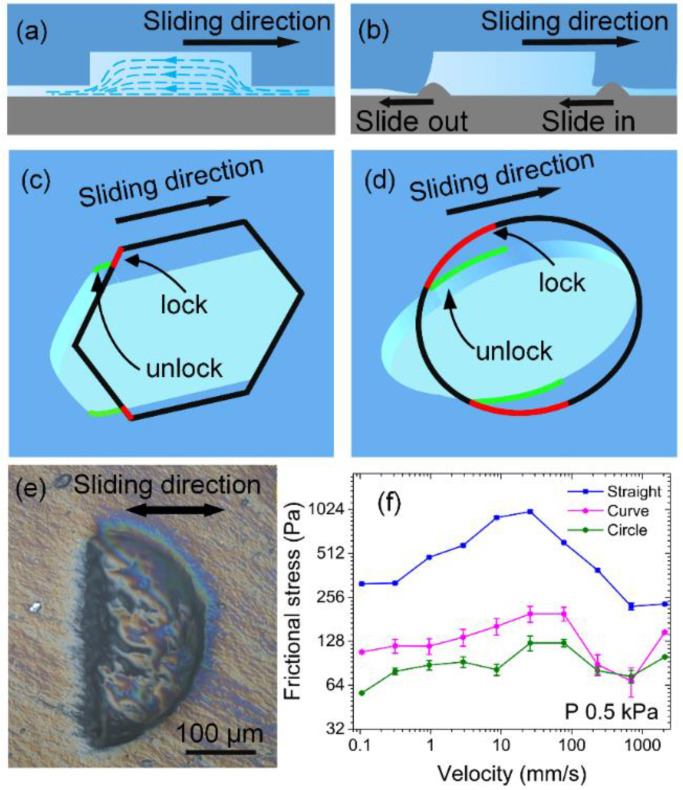
A schematic of the frictional-wear mechanism. (**a**) Fluid lubrication in a micro-pit. (**b**) Substrate micro-protrusions interlocking with a micro-pit. Deformation of (**c**) hexagonal and (**d**) circular micro-pits; black: micro-pit profiles before tensile deformation, red: trailing edges that would have interlocked before deformation, green: trailing edges that would haven’t interlocked with micro-protrusions on the substrate after deformation have been transformed from red profiles. (**e**) A confocal plot of a semicircular pit. (**f**) Frictional stress on a semicircular profile.

Based on the wear, models of micro-pit wear and drag reduction mechanisms were proposed, as shown in Figure 6. Both the surface area and the friction generated by the substrate micro-protrusions plowing over the surface are the same for hydrogels with or without micro-pits. During friction, the water in the micro-pits continuously provides lubrication for the frictional interfaces (Figure 6a). Each micro-pit has the same lubrication effect because of the same area and depth. The difference in friction originated from the interlocking friction between the substrate micro-protrusions and the micro-pits on the hydrogel surface. Figure 6b indicates that when the micro-protrusions slid from the smooth surface into the micro-pits, the plowing occurs only on the smooth surface, and it is easy for micro-protrusions to enter into the micro-pits. By contrast, the substrate micro-protrusions must overcome the strong interlock with the contour profile at the bottom of the micro-pits to slide out of them, which is consistent with the frictional-wear results.

The interlocking between trailing edges of different shapes and the substrate micro-protrusions caused micro-pit tensile deformation, which changed the length of the trailing edges that subsequently interlocked with the micro-pits. The triangular and quadrilateral trailing edges were perpendicular to the sliding direction and could not be pulled parallel to it when stretched by friction. The hexagonal and circular trailing edges were at an angle of <90° to the sliding direction. During the sliding and stretching, the red curves at two ends of the trailing edges were pulled to green curves, which were parallel to the sliding direction (Figure 6c,d). This process effectively reduced the length of the trailing edge that interlocked with the substrate micro-protrusions in the projection perpendicular to the sliding direction, particularly in the circular micro-pits.

To further verify that a change in the shape of the trailing edge makes a difference in friction, we tested friction on semicircular micro-pits with straight and curved trailing edges, respectively (Figure 6e). The results showed that the curved trailing edge has lower frictional stress than the straight one. Because semicircular micro-pits provide less aqueous lubrication owing to smaller volume, when the trailing edge is curved, semicircular micro-pits yield slightly greater friction than circular micro-pits do.

### 3.3. Modulation of Frictional Properties of Cartilaginous and Artificial Joints

**Figure 7:**
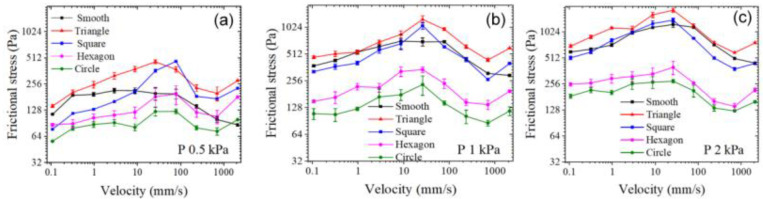
Frictional-stress variation on hydrogel surfaces containing different micro-pits when the titanium alloy served as the substrate. (**a**) P = 0.5 kPa, (**b**) 1 kPa, and (**c**) 2 kPa.

After a preliminary understanding of the mechanism of micro-pits in laser textures, we try to simulate the friction between cartilage and bone with hydrogel and titanium alloy as friction pairs. Figure 7 shows how the frictional stress varied with velocity on various micro-pit hydrogel surfaces when the titanium alloy served as the substrate under contact pressure. Both the smooth surface hydrogel and the micro-pit surface hydrogel obeyed the law of frictional-stress change with velocity in the repulsion–adsorption model. vf is the characteristic velocity of hydrogel with solid as friction substrate. When the sliding velocity was sufficiently small (v << vf), the frictional force was caused by the elastic force of the stretched polymer chains and increased with sliding velocity. A transition from elastic force to fluid lubrication began when sliding velocity (v) was approximately equal to vf. When sliding velocity was sufficiently large (v >> vf), the friction force primarily derived from viscous dissipation of the hydration layer and was proportional to sliding velocity [19].

Under contact pressure P of 0.5 kPa, frictional stress on the triangular micro-pit surface was the highest (143–463 Pa) throughout the sliding-velocity range. Frictional stress on the quadrilateral micro-pit surface (77–469 Pa) began to exceed frictional stress on the smooth-surface when v > 8.7 mm/s. Frictional stress was the lowest for hexagonal (87–197 Pa) and circular (56–124 Pa) micro-pit surfaces, and in both cases, was lower than smooth-surface frictional stress.

As contact pressure increased, frictional stress could still be ranked as “σ (triangle) > σ (quadrilateral) > σ (hexagon) > σ (circle),” whereas smooth-surface frictional stress increased more than that on the micro-pitted hydrogel surface. At P = 0.5 kPa with v > 8.7 mm/s, frictional stress was significantly higher on the triangular- and quadrilateral-micro-pit surfaces than on the smooth hydrogel surface (Figure 7a). On the contrary, as contact pressure P increased to 1 kPa, frictional stress on the smooth hydrogel surface increased to 295–717 Pa, which began to overlap with frictional stresses on the triangular-micro-pit (460–1258 Pa) and quadrilateral-micro-pit (265–1065 Pa) surfaces (Figure 7b). As P increased to 2 kPa, frictional stresses on the smooth hydrogel surface became higher than those on the triangular- and quadrilateral-micro-pit surfaces at some velocities (Figure 7c). As contact pressure increased from 0.5 to 2 kPa, maximum frictional stress on the hexagonal- and circular-micro-pit surfaces increased only slightly to 141–399 and 125–276 Pa, respectively. The fluid in the chamber (formed by micro-pits and the titanium alloy) shared the contact pressure, as was evident gradually with the increasing contact pressure (Figure 6a). In contrast, the random roughness or micro-protrusions studied previously have been connected with the outside via formation of a drainage channel, thereby preventing the water between the micro-structures of hydrogels and solid surfaces from bearing contact pressure.

**Figure 8:**
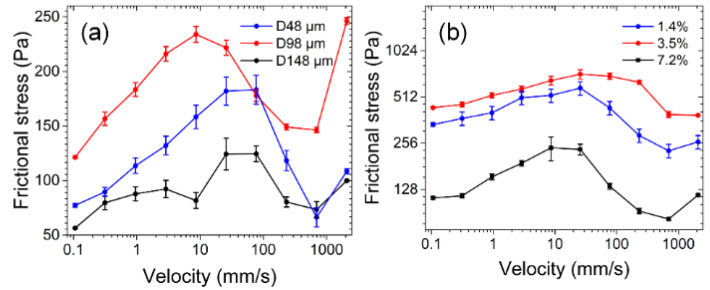
Effects of the diameter and porosity of circular micro-pits on frictional stress at the hydrogel–titanium alloy interface. micro-pit diameter (**a**) and porosity (**b**).

Figure 8a presents the friction tests of circular micro-pits with diameters of 48, 98, and 148 μm under 0.5 kPa contact pressure. As the diameter of the circular micro-pits gradually increased, frictional stress initially increased from 66–183 Pa to 121–234 Pa and then declined to 56–124 Pa. Similarly, adjusting porosity can also regulate frictional stress. Porosity was varied by adjusting the number of micro-pores with a diameter of 148 μm on the hydrogel surface and a test was conducted under a contact pressure of 1 kPa (Figure 8b). Frictional stress increased from 228–584 to 393–720 Pa and then decreased to 82–239 Pa when porosity increased from 1.4% to 7.2%, consistently with the law governing the relationship between the diameter and frictional force. The influence of micro-pit diameter and porosity on friction can be understood by the competition between (i) the contribution of the interlocking force of a micro-protrusion on the microporous titanium alloy to friction and (ii) the lubrication caused by the fluid on the alloy. Friction initially increased and then diminished with the increasing diameter and porosity.

### 3.4. Regulation of Frictional Properties between Cartilage Surfaces (Within an Experimental Joint)

**Figure 9:**
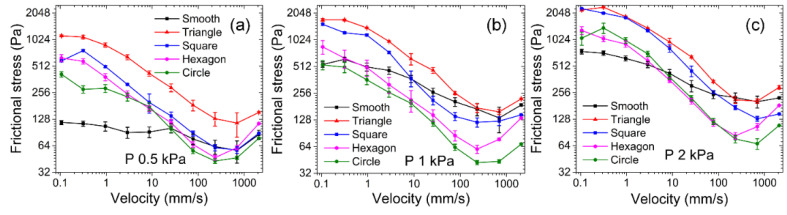
Variation of frictional stress on hydrogel surfaces containing micro-pits of different shapes with the hydrogels serving as substrates. (**a**) P = 0.5 kPa, (**b**) 1 kPa, and (**c**) P = 2 kPa.

Next, we used two hydrogels as friction pairs to simulate the friction behavior between cartilages. Figure 9 shows that frictional stress varied with velocity when hydrogel surfaces containing micro-pits of different shapes were subjected to different contact pressures. At the minimal tested velocity of 0.1 mm/s, the hydrogel–hydrogel interface entered a state of transition from elastic force to fluid lubrication, and frictional stress still obeyed the law of frictional-stress change with velocity in the repulsion–adsorption model.

The effect of micro-pits of different shapes on friction was consistent with that when the titanium alloy was the substrate. The ranking “σ (triangular) > σ (quadrilateral) > σ (hexagonal) > σ (circular)” was still correct.

At light loads (P = 0.5 kPa) and low velocities (0.1–10 mm/s), the smooth hydrogel provided good lubrication by retaining water between the interfaces. In contrast to the micro-pitted hydrogel surface, the smooth surface also maintained frictional stress in a low range (90–117 Pa) at low velocities. The ranking is subject to “σ(triangular) > σ(quadrilateral) > σ (hexagonal) > σ (circular)” until the velocity reaches 232 mm/s, which can be carefully identified in Figure 9a. In other words, the steady lubrication stage ranges from 0.1–232 mm/s. However, when the velocity is greater than 232 mm/s, the friction interface enters the stage of fluid lubrication, and the friction force begins to become unstable, thus the data fluctuation happens and a regularity “σ (triangular) > σ (quadrilateral) = σ (hexagonal) = σ (circular) = σ (smooth)” was observed. As contact pressure increased to 1 kPa, the micro-pit fluid bore a gradually greater proportion of contact pressure, and the increase of contact pressure on the smooth hydrogel significantly exceeded that on the micro-pitted hydrogel surface. At P = 2 kPa and v = 207 to 233 mm/s, frictional stress on the smooth hydrogel even exceeded that on the triangular-micro-pit–containing the hydrogel.

Under the test conditions of hydrogels as the substrate, the velocity of the interface entering the fluid lubrication state (v = 232 mm/s; Figure 9a,b) was lower than that when the titanium alloy served as the substrate (v = 696 mm/s; Figure 8). As contact pressure P increased to 2 kPa, the velocity at which the transition to fluid lubrication took place returned to 696 mm/s (Figure 9c). This was caused by differences in the hydrophilicity or hydrophobicity of the substrate. In contrast to the hydrophobic nature of the titanium alloy surface (CA = 96.7°) (Figure 1), the superhydrophilicity of the hydrogel surface (CA = 9.8°) kept water at the hydrogel–hydrogel interface, thereby leading to a decrease in adsorption points at low velocities and lower fluid lubrication velocity at high velocities.

### 3.5. Customized Design of Cartilage Joints

Artificial joint replacement surgery has become an important treatment for joint diseases worldwide, and hydrogels have emerged as a perfect substitute for articular cartilage owing to the similarity of their tribological properties to those of natural cartilage. Nonetheless, naturally, artificial joints have differences in materials and structure from joints in the human body. These circumstances require rational and selective design of artificial-cartilage performance, especially friction performance, to meet the requirements of different pathological conditions. Structure and constituent materials differ substantially between artificial and human joints. Moreover, the internal environments of joints are inconsistent among patients. In particular, a large cartilage functions in a complex environment, where it simultaneously is in contact with and rubs against multiple surfaces, resulting in significant differences between the sites of articular-cartilage wear [28,29]. Therefore, the rational and selective design of artificial cartilage, particularly in terms of frictional properties, is necessary to adapt this cartilage to friction with various surfaces, prolong cartilage life, and improve its fitness quality.

**Figure 10:**
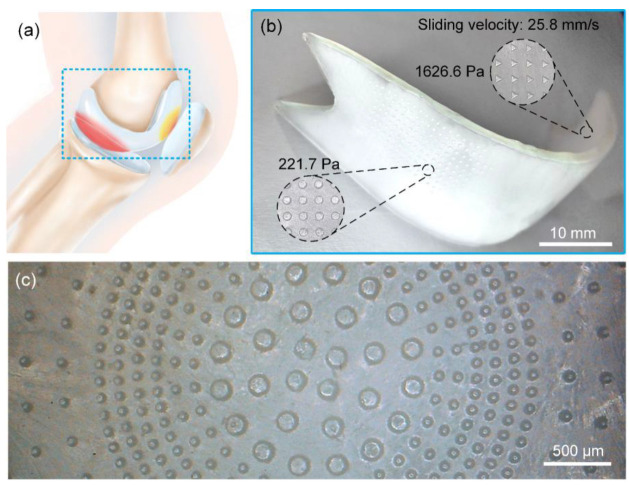
Rational selective design and application of hydrogel surface micro-pits. (**a**) Frictional-wear differences in knee cartilage. (**b**) A double-sided heterogeneous hydrogel. (**c**) A one-sided hydrogel with different sizes of circular micro-pits.

Human cartilage functions in a complex environment, with specific areas of articular cartilage facing a changing frictional working environment. Figure 10a shows that lower femoral cartilage forms different friction pairs with upper tibial and patellar cartilages featuring different frictional conditions. Frictional-wear conditions also vary among different sites within the same frictional pair. Therefore, the traditional one-shot molding method cannot ensure rational effective design. In this study, we rationally and selectively designed materials to meet the requirements of the complex environment where artificial joints should function. Figure 10b shows a hydrogel carrying triangular micro-pits featuring high friction on the upper surface and circular micro-pits offering low friction on the lower surface. Under a contact pressure of 2 kPa and a sliding velocity of 25.8 mm/s, frictional stress between the upper surface and titanium interface (1626.6 Pa) was 7.3 times that between the lower surface and hydrogel interface (221.7 Pa). Significantly, the frictional properties of each contact point can be precisely adjusted according to the differential wear of cartilage caused by regular physical exercise and according to its frequency to ensure that an artificial joint has the desired service life (Figure 10c).

## 4. Conclusions

At present, the research on the mechanism underlying the tribological properties of regular textured hydrogel surfaces on different substrates is not systematic and detailed. We utilized a UV laser to directly etch micro-pits of various shapes on PVA hydrogels and studied how they affected the tribological properties of the hydrogels. We drew the following conclusions:

When a hydrophobic titanium alloy surface served as the substrate, frictional-stress curves of micro-pitted and smooth hydrogels were both consistent with the law of frictional-stress change with velocity in the repulsion–adsorption model. Compared with this friction substrate, the superhydrophilic hydrogel friction substrate had a better ability to stably keep a fluid between the friction interfaces during low-speed sliding and to reduce the velocity at which the transition to fluid lubrication can take place.

On both substrates, the order “σ (triangular) > σ (quadrilateral) > σ (hexagonal) > σ (circular)” always held due to the frictional deformation of the micro-pits. As contact pressure increased, the rising trend of the friction stress curve of the smooth hydrogel became greater than that of the friction-stress curve of the micro-pitted hydrogel. These findings indicate that water in micro-pits can share pressure between the interfaces. Further experiments with the regulation of frictional stress by means of circular micro-pits revealed that frictional stress initially increases and then deceases with increasing diameter and porosity.

In addition, we demonstrated outstanding advantages of this method for the profiled design of artificial cartilage. Our results revealed the patterns of frictional-stress change with changes in velocity on hydrogel surfaces containing different micro-pit structures with different substrates and loads. These data are important for the development of biofriction systems and smart mechanical friction pairs.

## Figures and Tables

**Table 1:** Process parameters for laser-based fabrication of hydrogel surface micro-pits.

Laser Parameter	Symbol (Units)	Values
Average power	P(W)	1.88
Wavelength	λ(nm)	355
Pulse duration	τ(ns)	20
Repetition rate	F(kHz)	50
Spot diameter	φ(μm)	20

## Data Availability

The data presented in this study are available on request from the corresponding author.

## Supplementary Materials

The following are available online at https://www.mdpi.com/article/10.3390/nano12224103/s1, Section S1. Materials and Method, Figure S1. Schematics of the friction test and shear stress distribution. (A) The friction test device. (B) The schematic of the friction test device. (C) Frictional stress distribution. ρ, radial distance; σ_ρ_, frictional stress at a radial distance of ρ; σ_max_, maximum frictional stress; T, friction torque, Figure S2. Stress-strain curve of PVA hydrogel (Not displayed in the manuscript).
