# Supplementary material for: Modification of Frictional Properties of Hydrogel Surface via Laser Ablated Topographical Micro-Textures
Source: Nanomaterials (Basel). 2022 Nov 21;12(22):4103. doi: 10.3390/nano12224103 (PMC9696626; doi:10.3390/nano12224103)
Supplement: Supplementary file 1 [file nanomaterials-12-04103-s001.zip › nanomaterials-1967856-supplementary.pdf]

Supplementary Materials

Modification of Frictional Properties of Hydrogel Surface via Laser Ablated Topographical Micro-Textures

Zhuangzhuang Zhou [1,†], Yihang Chu [2,†], Zhishan Hou [1,*], Xiaopeng Zhou [1,2] and Yu Cao [1,3,*]

[1] International Science and Technology Cooperation Base for Laser Processing Robot, Zhejiang Provincial Key Laboratory of Laser Processing Robot, Wenzhou University, Wenzhou 325035, China
[2] School of Optical and Electronic Information, Huazhong University of Science and Technology, Wuhan 430074, China
[3] Oujiang Laboratory (Zhejiang Lab for Regenerative Medicine, Vision and Brain Health), Wenzhou University, Wenzhou 325000, China
* Correspondence: houzs21@wzu.edu.cn (Z.H.); yucao@wzu.edu.cn (Y.C.)
† These authors contributed equally to this work.

S1. Materials and Methods

S1.1. Depth Calculation Method

The arithmetic mean height of the sample surface (*Sa*) was determined by measuring and calculating the observed area of the sample. S_a is the arithmetic or geometric mean of the distance between points on the contour surface and the center surface in the observation area. The mathematical expression of S_a is as follows:

$$S_a = \frac{1}{l_x l_y}\int_0^{l_x}\int_0^{l_y}|Z(x,y)|dxdy \quad (1)$$

where l_x and l_y are the lengths of the region observed along the directions of the x-axis and y-axis, respectively. $Z(x,y)$ is the distance from the point on the contour surface with coordinates (x,y) to the center surface.

S1.2. Detailed Process of Friction Test

The double-sided adhesive was placed on the other side of the acrylic disk, which was then positioned in the center of a rotor using a customized positioning slot. A 3-mm-thick acrylic ring with outer and inner diameters of 50 and 45 mm was bonded to the surface of smooth titanium alloy disks or hydrogels to form titanium alloy and hydrogel friction substrates that were then fixed to the aluminum plate of a rheometer.

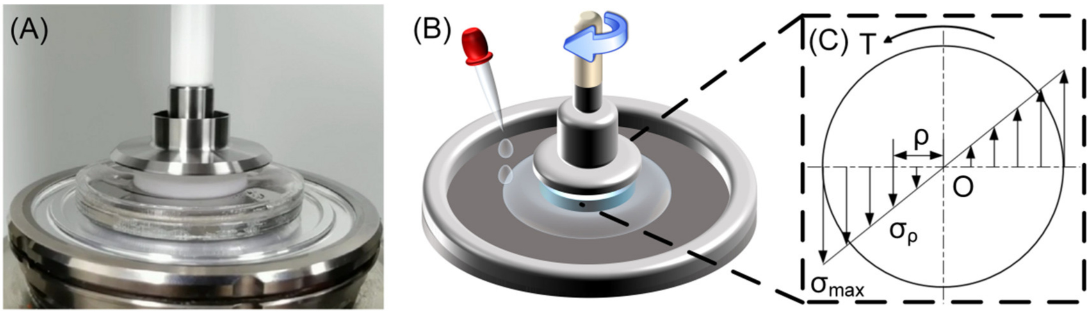

Figure S1. Schematics of the friction test and shear stress distribution. (**A**) The friction test device. (**B**) The schematic of the friction test device. (**C**) Frictional stress distribution. ϱ, radial distance; σ_ϱ, frictional stress at a radial distance of ϱ; σ_{max}, maximum frictional stress; T, friction torque.

The two interfaces were immersed in water (2ml, injected via a syringe) in a customized device to characterize the friction with the micro-pits of various shapes (Figure S1). Test velocity was 0.1–2,086.5 mm/s, contact pressure (P) was 0.5, 1.0, or 2.0 kPa. The hydrogel surface was controlled by means of a HAKKE MARS60 rheometer (Thermo Fisher Scientific Inc., Waltham, MA, USA) for vertical downward movement until contact pressure reached the preset value,

which was then maintained for 5 s. Extrusion was continued for 15 min under limited strain, so that the contact stress between the two interfaces remained stable during the test. After 1 min of prerotation at a sliding velocity of 2.6 mm/s, friction was assessed at different velocities. Real-time moment values were determined every 0.5 s. The torque at each velocity was measured and then immediately assessed at the next velocity, and there was no need for interface separation and reloading. The purpose of the prerotation was to prevent the hydrogel from undergoing only shear deformation during low-speed rotation, when the applied torque could not exceed static friction of the interface. The shear stress inside the hydrogel caused by the prerotation allowed the interface to reach the sliding-friction threshold sooner. Therefore, the duration of the test was longer in the low-speed range and shorter in the high-speed range (the duration was 540, 180, 60, and 30 s at $v = 0.1$, 0.3 to 2.9, 8.6 to 232.1, and 695.6 to 2086.5 mm/s, respectively). During the first 10 s, the rheometer was in the acceleration phase, and feedback torque did not match the required torque value. Friction torque $T(\omega)$ was assumed to be the average of the torque after 10 s of each test.

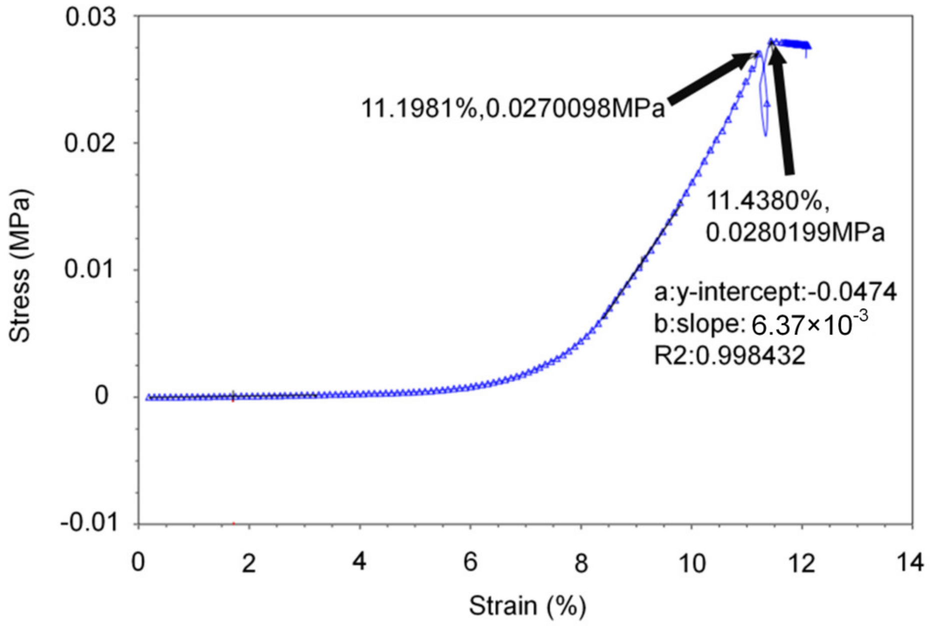

Figure S2. Stress-strain curve of PVA hydrogel (Not displayed in the manuscript).
